# Evaluation of high-dose daptomycin for therapy of experimental *Staphylococcus aureus *foreign body infection

**DOI:** 10.1186/1471-2334-6-74

**Published:** 2006-04-11

**Authors:** Heinz J Schaad, Manuela Bento, Daniel P Lew, Pierre Vaudaux

**Affiliations:** 1 Service of Infectious Diseases, Geneva University Hospital, 24 rue Micheli-du-Crest, CH-1211 Geneva 14, Switzerland; 2 Spital Interlaken, Weissenaustrasse 27, 3800 Unterseen, Switzerland

## Abstract

**Background:**

Daptomycin is a novel cyclic lipopeptide whose bactericidal activity is not affected by current antibiotic resistance mechanisms displayed by *S. aureus *clinical isolates. This study reports the therapeutic activity of high-dose daptomycin compared to standard regimens of oxacillin and vancomycin in a difficult-to-treat, rat tissue cage model of experimental therapy of chronic *S. aureus *foreign body infection.

**Methods:**

The methicillin-susceptible *S. aureus *(MSSA) strain I20 is a clinical isolate from catheter-related sepsis. MICs, MBCs, and time-kill curves of each antibiotic were evaluated as recommended by NCCLS, including supplementation with physiological levels (50 mg/L) of Ca^2+ ^for daptomycin. Two weeks after local infection of subcutaneously implanted tissue cages with MSSA I20, each animal received (i.p.) twice-daily doses of daptomycin, oxacillin, or vancomycin for 7 days, or was left untreated. The reductions of CFU counts in each treatment group were analysed by ANOVA and Newman-Keuls multiple comparisons procedures.

**Results:**

The MICs and MBCs of daptomycin, oxacillin, or vancomycin for MSSA strain I20 were 0.5 and 1, 0.5 and 1, or 1 and 2 mg/L, respectively. In vitro elimination of strain I20 was more rapid with 8 mg/L of daptomycin compared to oxacillin or vancomycin. Twice-daily administered daptomycin (30 mg/kg), oxacillin (200 mg/kg), or vancomycin (50 mg/kg vancomycin) yielded bactericidal antibiotic levels in infected cage fluids throughout therapy. Before therapy, mean (± SEM) viable counts of strain I20 were 6.68 ± 0.10 log_10 _CFU/mL of cage fluid (n = 74). After 7 days of therapy, the mean (± SEM) reduction in viable counts of MSSA I20 was 2.62 (± 0.30) log_10 _CFU/mL in cages (n = 18) of daptomycin-treated rats, exceeding by >2-fold (*P *< 0.01) the viable count reductions of 0.92 (± 0.23; n = 19) and 0.96 (± 0.24; n = 18) log_10 _CFU/mL in cages of oxacillin-treated and vancomycin-treated rats, respectively. Viable counts in cage fluids of untreated animals increased by 0.48 (± 0.24; n = 19) log_10 _CFU/mL.

**Conclusion:**

The improved efficacy of the twice-daily regimen of daptomycin (30 mg/kg) compared to oxacillin (200 mg/kg) or vancomycin (50 mg/kg) may result from optimisation of its pharmacokinetic and bactericidal properties in infected cage fluids.

## Background

Infections due to *Staphylococcus aureus *associated with foreign implants, such as orthopaedic prostheses and intravascular devices, are very difficult to manage by antimicrobial therapy alone and frequently require the removal of infected materials [1]. The growing proportion of clinical isolates of methicillin-resistant *S. aureus *(MRSA) displaying multidrug resistance not only against all semi-synthetic penicillins and penems, but also frequently against macrolides, aminoglycosides, fluoroquinolones [2-4], and glycopeptides [3,5-7] recently prompted the development of novel antimicrobial agents active against such dangerous pathogens.

Among recently developed agents overcoming antibiotic resistance in MRSA but also highly active against methicillin-susceptible staphylococci, the lipopeptide daptomycin [8-11] was already discovered in the 1980s by Lilly Research Laboratories that stopped its clinical development in 1991 due to reports of potential skeletal muscle toxicity. In 1997, daptomycin development was resumed by Cubist Pharmaceuticals [12] and its clinical use approved by FDA in 2003 for the treatment of complicated skin and soft tissue infections (CSSSIs) [11,13]. Daptomycin is known to exhibit calcium-dependent binding to bacterial cytoplasmic membranes which leads to disruption of membrane function [14]. Daptomycin is uniformly potent in vitro against *S. aureus *clinical isolates in large surveillance studies [11,15-18]. An interesting property of daptomycin is its rapid bactericidal activity in vitro [9,12,19,20] and in vivo [9,21-23], but this bactericidal activity is concentration-dependent and its optimal expression may sometimes require levels equivalent to 8-fold the lipopeptide MIC for a given strain [12,19]. The relatively high protein binding and low volume of distribution of daptomycin recorded in humans or animal models represent a difficult challenge for defining a dosing schedule exerting optimal bactericidal activity against major categories of serious *S. aureus *infections [12,21-29] in various deep-seated compartments though minimizing skeletal muscle side-effects [30,31]. Another issue is the recently shown observation that pulmonary surfactant interfered with daptomycin antimicrobial activity thus providing a likely explanation for treatment failures in clinical trials of community-acquired pneumonia and animal models of gram-positive pneumonia [28].

We previously described an animal model for evaluating the in vivo activity of various bactericidal antibiotics against localized chronic foreign body infections due to *S. aureus *[32-38]. This model consists in perforated Teflon cylinders, named tissue cages, which are subcutaneously implanted in rats and infected three to four weeks after surgical implantation by percutaneous local inoculation of 10^5 ^CFU of methicillin-susceptible (MSSA) or methicillin-resistant (MRSA) strains of *S. aureus*, yielding sustained bacterial viable counts in tissue cage fluids for several weeks [32]. The presence of the inflammatory exudative fluid inside tissue cages, designated as tissue cage fluid (TCF), allows a direct assessment of the levels and pharmacokinetic properties of any antimicrobial agent that accumulates into this compartment after systemic administration. Drug efficacy is evaluated by comparing quantitative cultures of each cage fluid before and after 7 days of intensive antimicrobial therapy [32]. Several studies have shown that systemic administration of various categories of antibiotics can deliver adequate levels of antimicrobial agents in TCFs [32-36]. In those studies, the nearly continuous presence of bactericidal levels of each antimicrobial was required throughout the 7-day therapies to obtain a significant reduction of at least one log_10 _CFU of MSSA or MRSA per ml of TCF, as shown with vancomycin [32], oxacillin and imipenem [33], and several fluoroquinolones including sparfloxacin and temafloxacin [35], levofloxacin and trovafloxacin [36]. With respect to the low efficacies of 7-day monotherapies with previously tested antimicrobial agents against *S. aureus *chronically infecting tissue cages, the concentration-dependent, highly bactericidal activity of daptomycin seemed to be promising in this model.

A first study (sponsored by Lilly and referred to as the daptomycin no 1-study) was initiated in 1991 for evaluating daptomycin in the rat model of tissue cage infections. The study protocol was composed of two arms, the first one assessing in vivo activity of daptomycin and comparators against MSSA-infected and the second one against MRSA-infected tissue cages. Because of concern with potential reduction of drug efficacy due to high protein binding, a twice-daily regimen of 30 mg/kg daptomycin was selected for the daptomycin no 1-study, which resulted in very high levels of daptomycin in TCFs and led to impressive reductions in cage fluid viable counts in the MSSA arm that was the only one to be completed. In contrast, the MRSA arm of the daptomycin no 1-study was interrupted by Lilly's decision to suspend the development and experimental testing of daptomycin, which left data from the daptomycin no 1-study essentially unpublished (excepting for a brief report) [39].

Following resumption of daptomycin clinical development by Cubist in 1997, we initiated a second study (referred to as the daptomycin no 2-study) using a modified protocol that took into consideration the lower toxicity of once-daily administration of daptomycin [30,31]. With respect to the very high levels recorded in TCFs during the daptomycin no 1-study, we anticipated that a 50% reduction in drug dosage, consisting in a single daily administration of 30 mg/kg daptomycin, would still deliver high, continuously bactericidal drug levels for therapy in *S. aureus*-infected cage fluids. Unexpectedly, the TCF levels recorded with the once-daily regimen of 30 mg/kg daptomycin turned out to be much lower than those anticipated from the 50% reduction of the previously used twice-daily regimen administered in the daptomycin no 1-study [37]. While the drug levels reached in TCFs by the once-daily 30 mg/kg daptomycin regimen were still over the minimal bactericidal concentration and led to significant viable count reductions in *S. aureus*-infected cage fluids [37], those drug levels were clearly inferior to the 8-fold levels over the MIC of the infecting organism recommended for optimal daptomycin bactericidal activity against *S. aureus *[8,9,12,21].

In the context of recently presented pharmacological, experimental, and clinical studies exploring the safety and efficacy of higher once-daily doses of daptomycin [29,40-42] for treating serious gram-positive infections (e.g. *S. aureus *bacteremia, endocarditis, osteomyelitis), compared to the FDA-approved 4 mg/kg daily regimen for complicated skin and skin-structure infections (CSSSIs), the presentation of pharmacokinetic and efficacy data obtained during the daptomycin no 1-study gains increased relevance. The aims of this report are to (i) show the impact of the twice-daily 30 mg/kg daptomycin regimen in the therapy of chronically infected tissue cages compared to oxacillin and vancomycin; (ii) emphasize the major differences in TCF drug levels reached by administration of a twice-daily compared to once-daily 30 mg/kg daptomycin regimen [37]; (iii) discuss the pharmacokinetic and pharmacodynamic factors that may contribute to daptomycin efficacy in our hard-to-treat rat tissue cage model of foreign body infections as well as other infection models or clinical situations.

(An abstract form of this study was presented at the 42nd Intersci. Conf. Antimicrob. Agents Chemother., abstr. 1904, 2002) [39].

## Methods

### Bacterial strain

The methicillin-susceptible *S. aureus *(MSSA) strain I20 is a previously described clinical isolate from catheter-related sepsis, which is highly virulent in the rat model of chronic *S. aureus *tissue cage infection [33,43]. Strain I20 is uniformly susceptible to all antibiotics except penicillin.

Besides the strain used for the animal study, a previously mentioned collection of 107 *S. aureus *clinical strains isolated before 1991 in the University Hospital of Geneva [33] were also tested for in vitro susceptibility to daptomycin. They comprised 57 MSSA and 50 MRSA strains.

### Antimicrobial agents

All experimental in vitro and in vivo results reported in the daptomycin no 1-study, also including the 107 clinical isolates tested for in vitro susceptibility, were recorded in 1991 using a single batch of quality-controlled daptomycin powder supplied by Ely Lilly (Giessen, Germany). Daptomycin solutions were freshly prepared in distilled sterile water, namely at a concentration of 5 mg/mL for in vitro studies and 7.5 mg/L for in vivo studies. Oxacillin (Sigma Chemical, St. Louis, Mi) was freshly dissolved in distilled water at a concentration of 20 mg/ml. Commercially available vancomycin hydrochloride (Lilly) was solubilized as recommended by the manufacturer.

### In vitro studies

The MICs of oxacillin and vancomycin for strain I20 were determined in cation-adjusted Mueller-Hinton broth (CAMHB) containing 20-25 mg/L Ca^2+ ^and 10-12.5 mg/L Mg^2+ ^by the standard tube macrodilution method with an average inoculum of 10^6 ^CFU/mL as described by the Clinical Laboratory Standards Institute (CLSI) [44]. For daptomycin MIC, CAMHB was supplemented with additional calcium to a physiological concentration of 50 mg/L Ca^2+ ^(CSMHB).

To screen for the possible carryover effects of each antibiotic during the MBC determinations, 0.1-mL portions were taken from all tubes with no visible growth. These were subcultured, either undiluted or diluted 10-fold in saline, on Mueller-Hinton agar (MHA) for 36 h at 37°C. The MBC was defined as the lowest concentration that killed 99.9% of the original inoculum.

Each MIC and MBC determination was performed three times and the modal values are presented. In additional control experiments, MICs and MBCs were performed in CAMHB supplemented with 50% TCF.

### Killing kinetic studies

0.1-mL portions containing 10^6 ^CFU of strain I20 (obtained from exponential-phase cultures) were added to sterile plastic tubes containing 1 mL of either CSMHB containing 8 mg/L of daptomycin, or CAMHB containing 8 mg/L of oxacillin or vancomycin, in a shaking waterbath at 37°C. The number of viable organisms was determined by subculturing 50 μL of 10-fold diluted portions on MHA after 0, 2, 4, 6, and 24 h of incubation. Colonies were enumerated with a laser colony counter (Spiral System) after 48 h of incubation at 37°C. The detection limit was 2 log_10 _CFU/mL. No significant carryover of antibiotics was observed by using these experimental conditions. Each killing kinetic experiment was repeated three times and results from one representative experiment are presented.

### Treatment of chronic tissue cage infections

All animal studies of the daptomycin no 1-study were performed in 1991. These animal studies followed ethical guidelines of that time period and received legal approval from the Veterinary Office of the State of Geneva. Subcutaneous surgical implantation of four tissue cages per animal was performed as described [32] by using Wistar rats that were anaesthetised with an intraperitoneal (i.p.) injection of a mixture of ketamine (90 mg/kg) and xylazine (5 mg/kg). At 3 weeks post-implantation, TCF was aspirated and checked for sterility. To establish a chronic *S. aureus *infection, tissue cages were inoculated with 0.1 mL of saline containing 0.2 × 10^6 ^to 2 × 10^6 ^CFU of a washed overnight culture of strain I20 in CAMHB. Three weeks later, all tissue cages containing more than 10^5 ^CFU/mL of TCF were included in the therapeutic protocols.

Rats infected with strain I20 were randomised to receive (by the i.p. route for 7 days) twice-daily regimens of either daptomycin (30 mg/kg), oxacillin (200 mg/kg), or vancomycin (50 mg/kg), or were left untreated. At 12 h after the last injection of antibiotic, quantitative cultures of 10-fold serially diluted TCF were performed on MHA. To optimise the yield of viable bacteria, TCF were briefly sonicated (60 W, 1 min) to disrupt the biofilm and phagocytic cells before the serial dilutions and plating. Plates were incubated for 24-48 h at 37°C. The detection limit was one colony equivalent to 2 log_10 _CFU/mL of TCF. The differences in CFU counts between day 1 and day 8 were determined and expressed as delta log_10_CFU/mL. For each treatment group, results were expressed as means ± standard errors of the means (SEM). Comparison of bacterial counts in the different groups was performed by ANOVA and Newman-Keuls multiple comparisons procedures using Microsoft Excel^® ^2000 software [45]. Data were considered significant when *P *was < 0.05 by using two-tailed significance levels.

To check for potential side effects of the twice-daily 30 mg/kg daptomycin regimen, serum samples of the daptomycin-treated and control rats were collected at day 8 for determination of liver, kidney, and muscle enzyme markers, namely aspartate aminotransferase (ASAT) and alanine aminotransferase (ALAT) activities, serum creatinine, and creatine kinase, respectively, using standard analytical methods.

### Pharmacokinetics of antimicrobial agents

The pharmacokinetic levels of daptomycin were determined in TCF of uninfected rats, at various time intervals (0, 2, 4, 6, and 12 h) on day 4 and day 7 of twice-daily administration of 30 mg/kg daptomycin as described in the daptomycin no 2-study [37]. *S. aureus*-infected tissue cage fluids were not assayed to avoid potential interference with the daptomycin microbiological assay and minimize plasma contamination of TCF samples. Plasma daptomycin levels were determined on rat blood also collected from uninfected rats by periorbital puncture at various time intervals (0.25, 0.5, 1, 2, 4, 6, 8, and 12 h) after i.p. administration of this antibiotic. For each time point, the mean (± SEM) daptomycin levels in plasma from four animals was determined.

The concentrations of daptomycin in rat plasma or TCF were estimated by a previously described microbiological assay [46] excepting for the use of Antibiotic medium 11 and *Sarcina lutea *as the test strain [37]. To avoid a potential bias due to protein binding, all plasma or TCF samples were diluted with 1 volume of PBS and assayed in duplicate, with reference to a range of standard concentrations (1-128 mg/L) of daptomycin, also prepared in duplicate in PBS supplemented with 50% plasma or TCF, respectively. The correlation coefficients of daptomycin standard curves were generally ≥ 0.99, yielding intra-assay CVs of ± 10%. Under these experimental conditions, the limit of detection of the daptomycin assay was 1 mg/L. For each time point, the mean (± SEM) daptomycin levels in TCF from six animals were determined.

The areas under the concentration-time curve (AUC) of daptomycin in rat plasma or TCF were estimated from 0 to 12 h (AUC_0-12_) by the linear trapezoidal rule. For comparison with a previously reported AUC_0-24 _in the daptomycin no 2-study, under a once-daily 30 mg/kg daptomycin regimen [37], we multiplied the AUC_0-12 _estimated from the twice-daily daptomycin regimen by a factor of two.

The pharmacokinetic properties of oxacillin [33] and vancomycin [32] in rat tissue cage fluid were previously described.

## Results and discussion

### In vitro studies

The MIC and MBC of daptomycin in CSMHB for MSSA I20 were 0.5 and 1 mg/L, respectively. The MICs and MBCs of oxacillin or vancomycin in CAMHB for MSSA I20 were 0.5 and 1 or 1 and 2 mg/L, respectively.

The MICs of daptomycin for 107 clinical isolates tested with the same batch of daptomycin as used for the rat tissue cage study were very similar to those reported 10 years later using more recent daptomycin batches [11,15-18]. Daptomycin MICs were 0.5, 1, and 2 mg/L for 5, 98, and 4 isolates, respectively. These data assessed that the antimicrobial potency of daptomycin used in the daptomycin no 1-study was equivalent to that used 10 years later for the daptomycin no 2-study [37].

**Figure 1:**
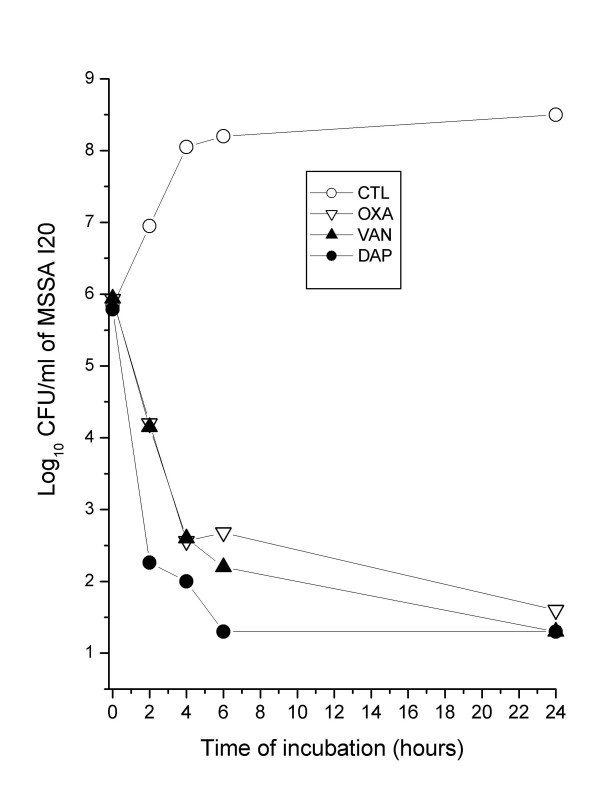
Elimination rates of methicillin-susceptible *S. aureus *(MSSA) strain I20 with daptomycin (DAP; 8 mg/L), oxacillin (OXA; 8 mg/L) or vancomycin (VAN; 8 mg/L), assayed in CSMHB (DAP) or CAMHB (OXA; VAN). These data are from a single representative experiment and were reproduced three times.

The in vitro elimination rate of exponentially grown MSSA I20 was more rapid with 8 mg/L of daptomycin than with equivalent concentrations of oxacillin or vancomycin (Figure 1). The reduction in the viable counts of strain I20 by daptomycin, oxacillin, and vancomycin exceeded 3 log_10 _CFU/mL after 2, 4, and 4 h, respectively. The more rapid bactericidal activity of daptomycin over oxacillin and vancomycin was reproduced in three independent experiments.

The elimination rate of MSSA I20 by daptomycin was not significantly affected by supplementation of CSMHB with 50% TCF (data not shown).

### Treatment of chronic tissue cage infections

At the onset of therapy, average bacterial counts for 74 cages infected with MSSA I20 were 6.85 ± 0.25 CFU/mL for controls (n = 19), 6.61 ± 0.19 log_10 _CFU/mL for animals receiving daptomycin (n = 18), 6.82 ± 0.20 log_10 _CFU/mL for animals receiving oxacillin (n = 18), and 6.46 ± 0.20 log_10 _CFU/mL for animals receiving vancomycin (n = 19). These four sets of bacterial counts did not differ significantly from one other.

**Figure 2:**
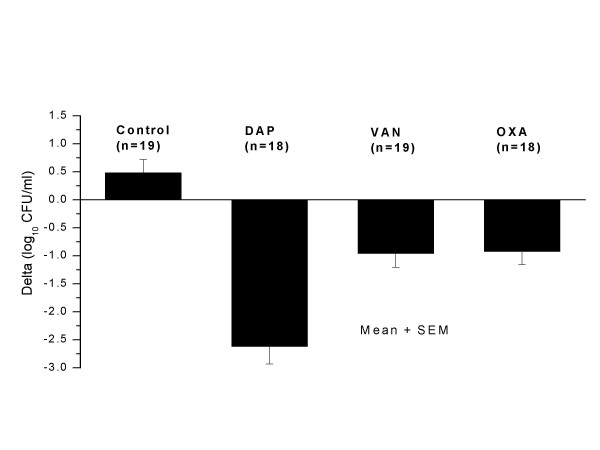
Decrease in viable counts of methicillin-susceptible *S. aureus *(MSSA) in tissue cage fluids of rats treated for 7 days with twice-daily regimens of either daptomycin (30 mg/kg), oxacillin (200 mg/kg), or vancomycin (50 mg/kg). n: number of cages included in each group.

At the end of the 7-day therapy, average counts in tissue cages of control, daptomycin-treated, oxacillin-treated, and vancomycin-treated animals were 7.33 ± 0.14 (n = 19), 3.99 ± 0.34 (n = 18), 5.90 ± 0.25 (n = 18), and 5.49 ± 0.21 log_10 _CFU/mL (n = 19), respectively. Compared to the control group that showed a slight albeit non-significant increase of 0.48 ± 0.24 log_10 _CFU/mL, all antibiotic regimens led to significant (*P *<0.001) reductions in bacterial counts in TCF, namely 2.62 ± 0.31 CFU/mL for animals receiving daptomycin, 0.92 ± 0.21 CFU/ml for animals receiving oxacillin, and 0.96 ± 0.24 log_10 _CFU/mL for animals receiving vancomycin (Figure 2). The mean reduction in CFU counts in TCF of rats treated with daptomycin was significantly (*P *< 0.01) higher (>2-fold) than that of oxacillin- or vancomycin-treated animals.

### Pharmacokinetic properties of daptomycin in plasma and tissue cage fluid

**Figure 3:**
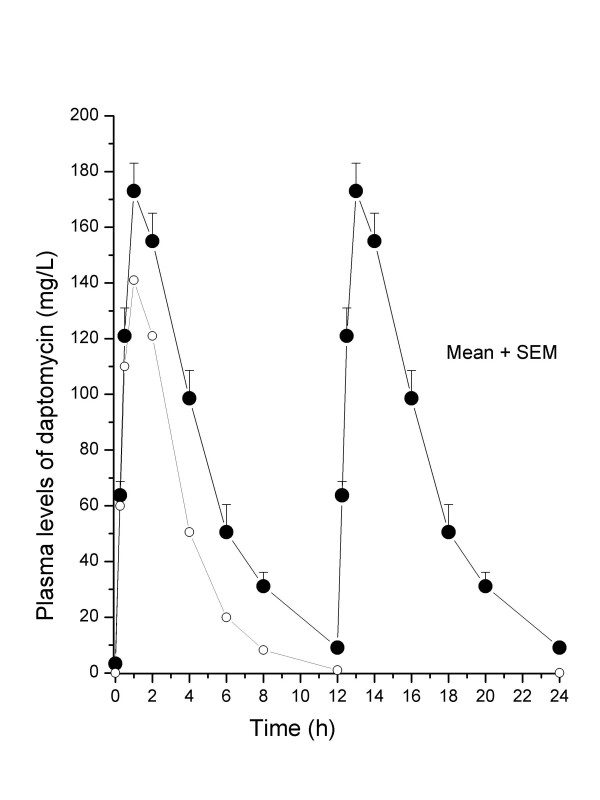
Pharmacokinetic levels of daptomycin in rat plasma on day 4 of therapy with twice-daily (closed symbols) or once-daily (open symbols) doses of daptomycin (30 mg/kg). The plasma AUC_0-24h _of rats treated with the twice-daily and once-daily regimens were 1667 and 558 mg × h/L, respectively. The values for each time point are the mean (+ SEM) daptomycin plasma levels determined in four different animals.

The pharmacokinetic levels of daptomycin in rat plasma on day 4 of daptomycin therapy are shown on Figure 3. On day 4 of administration with twice-daily doses (30 mg/kg) of daptomycin, mean peak and trough plasma levels were 173 and 9.1 mg/L at 1 h and 12 h, respectively. Similar concentrations of daptomycin were recorded on day 7 of therapy (data not shown). For comparison, the peak (141 mg/L) and trough (0 mg/L) plasma levels on day 4 of administration with once-daily doses (30 mg/kg) of daptomycin recorded in the daptomycin no 1-study [37] are represented on Figure 3. The pharmacokinetic profiles of twice-daily and once-daily regimens of daptomycin are not markedly different besides the much lower level recorded at 12 h for the once-daily (1 mg/L) compared to twice-daily (9.1 mg/L) dosing. The similar pharmacokinetic profiles obtained in both independently conducted studies using two different methods, namely a microbiological assay and HPLC, provide support to the reliability of these data and further indicate that the antimicrobial potency of daptomycin used for the daptomycin no 1-study was equivalent to that used 10 years later for the daptomycin no 2-study [37]. The plasma AUC_0-24h _(1667 mg × h/L) recorded with the twice-daily regimen was >2-fold higher than the once-daily regimen of 30 mg/kg daptomycin that yielded a plasma AUC_0-24h _of 558 mg × h/L [37].

**Figure 4:**
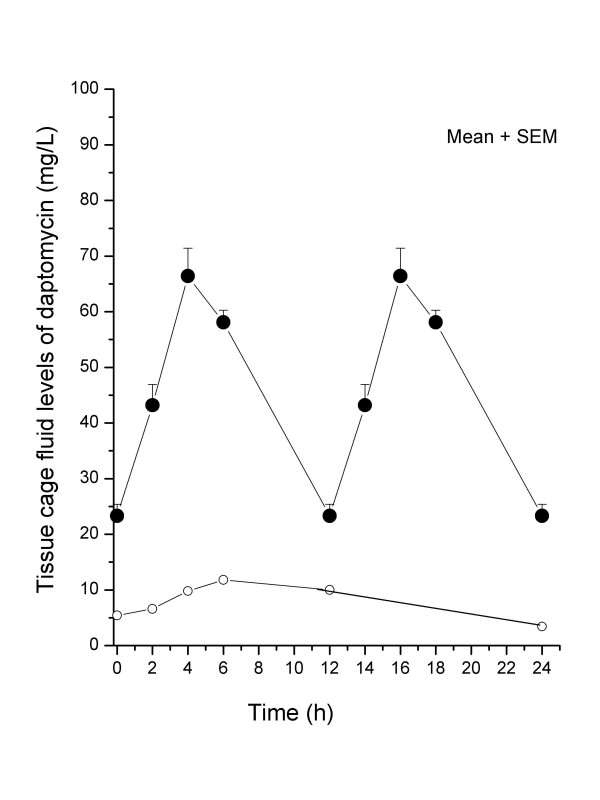
Pharmacokinetic levels of daptomycin in rat tissue cage fluids on day 4 of therapy with twice-daily (closed symbols) or once-daily (open symbols) doses of daptomycin (30 mg/kg). The tissue cage fluid AUC_0-24h_of rats treated with twice-daily and once-daily regimens of daptomycin were 1073 and 196 mg × h/L, respectively. The values for each time point are the mean (+ SEM) daptomycin TCF levels determined in six different animals.

More striking differences were observed when comparing pharmacokinetic levels of daptomycin reached in rat TCF by twice-daily versus once-daily administration of this antibiotic (Figure 4). Mean peak and trough levels of daptomycin in TCF were 66 and 21 mg/L for rats receiving the twice-daily daptomycin regimen. In comparison, mean peak and trough TCF levels of daptomycin were 11.8 and 3.4 mg/L for rats receiving the once-daily daptomycin regimens, with an intermediate value of 10.0 mg/L at 12 h. The most significant difference in pharmacokinetics was a ca. 5.5-fold increase in TCF AUC_0-24h _of rats receiving twice-daily (1073 mg × h/L) compared to once-daily (196 mg × h/L) regimens of daptomycin.

In rats receiving twice-daily doses of oxacillin (200 mg/kg) or vancomycin (50 mg/kg), peak and trough TCF levels were 45 and 5.7 or 12 and 2 mg/L, respectively, as described previously [32,33].

### Screening of potential side effects of high-dose daptomycin

No exterior sign of toxicity was observed on rats receiving twice-daily doses of daptomycin. There were no or only minor changes in activities of marker enzymes of daptomycin-treated (n = 5) vs control (n = 5) animals, namely 216 ± 24 vs 146 ± 36 IU/L for ASAT, 46 ± 9 vs 41 ± 11 IU/L for ALAT, 53 ± 5 vs 50 ± 5 μM/L for serum creatinine, and 1393 ± 886 vs 1349 ± 820 IU/L for creatine kinase, respectively. Thus, only mild liver toxicity was observed in rats treated with high-dose daptomycin.

### General comments

The improved efficacy of the twice-daily 30 mg/kg daptomycin regimen compared to oxacillin (200 mg/kg) or vancomycin (50 mg/kg) in the rat model of tissue cage infections by MSSA strain I20 should be discussed in the light of recent clinical and experimental studies that explored the safety and efficacy of higher regimens of daptomycin [29,40-42] against serious *S. aureus *infections (e.g. bacteremia, endocarditis, osteomyelitis) compared to the FDA-approved 4 mg/kg daily regimen for CSSSIs. The in vivo activity of the twice-daily 30 mg/kg daptomycin regimen in MSSA-infected cages was impressive when compared to the repeatedly low activity of the twice-daily 200 mg/kg regimen of the reference agent oxacillin recorded in this and a previous study [37]. While promising results were also recorded in daptomycin-treated rats infected with the MRSA strain MRGR3, the inadequately low numbers of control or vancomycin-treated animals prevented statistical validation of the MRSA arm (data not shown).

The more rapid in vitro elimination rate of MSSA strain I20 by daptomycin compared to oxacillin and vancomycin may be one factor potentially explaining its improved in vivo activity over comparators against tissue cage infections by MSSA strain I20. An additional contributing factor seems to be the very high TCF antibiotic levels resulting from the twice-daily 30 mg/kg daptomycin regimen. Nevertheless, a direct comparison between *S. aureus *in vivo elimination rates recorded in the daptomycin no 1- and -no 2 studies is not feasible and would require a correction factor accounting for the 2-fold difference in daptomycin MICs displayed by the different *S. aureus *strains used for those studies.

Despite the fact that current pharmacokinetic models do not adequately explain the widely different TCF levels recorded with the twice daily compared to once daily 30 mg/kg daptomycin regimens, we would like to propose the following interpretation. First, the plasma AUC_0-24h _of 558 mg × h/L estimated during the daptomycin no 1-study was considered slightly higher than the average AUC_0-24h _of human volunteers receiving a clinical once-daily dose of 4 mg/kg [37]. Since protein binding of daptomycin is comparable between rodents and humans, it has been argued that a comparison of total daptomycin concentrations is appropriate [47]. The plasma levels of the daptomycin no 1-study were also within the range of those recorded in a therapeutic rat model of MRSA endocarditis, which yielded plasma AUC_0-24h _of 278 and 605 mg × h/L following daily doses of 25 and 40 mg/kg of daptomycin, respectively [47]. In particular, the 40 mg/kg daptomycin daily dose in rats was considered slightly inferior to the 6 mg/kg daily dose administered to humans, which was recently shown to be efficient for therapy of *S. aureus *bacteremia and endocarditis [40]. Furthermore, another recent study demonstrated the necessity to administer 50 and 60 mg/kg twice-daily daptomycin regimens for efficient therapy of experimental osteomyelitis in rabbits [42]. In the same line, the safety and pharmacokinetics of higher daily regimens of daptomycin were also explored up to 8 mg/kg [29], and even more recently up to 12 mg/kg [41] in human volunteers.

Nevertheless, it should be stressed that it is not the plasma but the TCF AUC_0-24h _of daptomycin which is the true parameter directly influencing the therapeutic efficacy of this antibiotic at the local site of *S. aureus *infection. In the daptomycin no 1-study, the TCF AUC_0-24h _of daptomycin represented only 35% of that recorded in rat plasma, which indicated an incomplete diffusion of this antibiotic from the plasma to tissue cage fluid compartment when administered at the 30 mg/kg daily regimen [37]. In contrast, the TCF AUC_0-24h _of daptomycin administered at the 30 mg/kg twice-daily regimen in the daptomycin no 2-study was equivalent to 64% of that recorded in rat plasma. This result might be explained by either improved diffusion of this antibiotic from the plasma to TCF compartment or/and higher accumulation and slower elimination of this antibiotic in TCF compared to plasma. In this context, it should be mentioned that daptomycin pharmacokinetic parameters were shown to become non-linear at a 8 mg/kg daily regimen including a longer half-life of elimination in human volunteers [29]. It is therefore possible that pharmacokinetic data recorded when using the twice-daily 30 mg/kg daptomycin regimen in our rat model also reflected a non-linear pharmacokinetic response compared to the daptomycin no 1-study, which was further amplified in TCF compared to plasma compartment.

Elaborated pharmacokinetic studies in the immunocompromised murine thigh model have defined the AUC/MIC ratio (range 300-1000) as the pharmacodynamic parameter most predictive of this compound's bactericidal activity against *S. aureus *[21-23]. When applied to our rat model of tissue cage *S. aureus *infections, the AUC/MIC ratio of the daptomycin no 1-study would be 98 and that of the daptomycin no 2-study 1093, which could explain the improved bacteriological result of the twice-daily over once-daily 30 mg/kg daptomycin regimens. Nevertheless, application to the rat model of tissue cage infections of AUC/MIC ratio breakpoints recorded in neutropenic mice models is probably not justified because of important differences between these animal models. It is noteworthy that daptomycin therapy is initiated as early as 2 h post *S. aureus *infection in neutropenic mice, which may still be in the range of prophylactic therapies. In contrast, antimicrobial therapy in the tissue cage model is not initiated before 2 or 3 weeks of chronic *S. aureus *infections, which more closely mimic the clinical situation of foreign body infections [1]. Finally, the impact of protein binding is less clearly established in the TCF compared to plasma compartment, since we found no significant in vitro reduction of daptomycin bactericidal activity against MSSA by 50% TCF in this and the daptomycin no 1-study [37].

## Conclusion

In conclusion, our data further emphasise the value of performing experiments in animals for the primary evaluation of new therapeutic agents. Prediction of the therapeutic efficacies of various antibiotics against foreign body infections may be difficult by relying exclusively on in vitro pharmacodynamic models derived from pharmacokinetic data in the plasma compartment.

## Competing interests

The author(s) declare that they have no competing interests.

## Authors' contributions

HS and DL were involved in the study design. HS and MB performed the experimental study and acquisition of data. HS and PV performed data analysis and PV wrote the final draft of this paper. HS and DL provided input into subsequent drafts and iteration of this manuscript. All authors read and approved the final manuscript.

## Pre-publication history

The pre-publication history for this paper can be accessed here:


